# Ida B. Wells‐Barnett as an anticolonial theorist on crime and punishment

**DOI:** 10.1111/1468-4446.13179

**Published:** 2024-12-11

**Authors:** AunRika Tucker‐Shabazz, Veda Hyunjin Kim

**Affiliations:** ^1^ Department of Sociology University of Michigan Ann Arbor Ann Arbor Michigan USA; ^2^ Department of Sociology and Anthropology Ohio Wesleyan University Delaware Ohio USA

**Keywords:** anticolonial theory, crime, empire, Ida B Wells‐Barnett, lynching, punishment

## Abstract

Treasuring the legacy of Ida B Wells‐Barnett as a Black feminist is a vital liberatory commitment, as previous scholarship has commendably demonstrated. Equally important, however, is the need to present Wells‐Barnett as an anticolonial theorist whose scholarly texts—*Southern Horrors*, *A Red Record*, and *Crusade for Justice*—should be incorporated into social theory curricula. This article examines Wells‐Barnett's acute apprehension of the foundational structures of the US empire‐state in her scholarly writings on lynching. As she analysed, the white mob violence epitomised the co‐re‐formation of race and gender, rule of difference, and subversion of offender‐judge relationship. The agency of non‐state actors (e.g., lynch mobs) and government agents (e.g., judge and politicians) co‐constituted the reformation—not total transformation—of these foundational structures. Lynching, therefore, was the lynchpin of the US empire‐state in the post‐Reconstruction period: it sustained the white supremacist order by imposing a mass death penalty on Black people, while simultaneously serving as a disgrace to US civilization. To conclude, we highlight how Wells‐Barnett's theory offers broader relevance to anticolonial/postcolonial sociology, particularly through her subaltern standpoint, attention to the role of non‐state actors, and her commitment to intersectional analysis.

## 1 INTRODUCTION

Ida B Wells‐Barnett is a significant anticolonial theorist, especially concerning the US empire‐state (Glenn, 2015; Jung, 2015, p. 56) and its reformation into a mass punishment regime with the structural persistence of the racist order after the 1863 Emancipation. Her *scholarship* has been denied the status of such prominence (but, Agozino & Ducey, 2020; Puri, 2021), despite the recent movement to include postcolonial/decolonial/anticolonial thinkers in our list of the disciplinary canon. Her scholarly writings, that is, *The Southern Horror* and *A Red Record*, were published in 1892 and 1895, respectively, as pamphlets for the antiracism activists to freely make use of. She did not publish her book with a major press like AC McClurg & Co., nor did she earn PhD from Harvard, but the first sentence of *A Red Record* is resoundingly addressed to ‘[t]he student of American sociology’ (*ARR*: 71)^1^ of the late‐19th century. Alongside *Crusade for Justice*—Wells‐Barnett's autobiography that her daughter, Alfreda Duster, posthumously published in 1970—the two aforementioned books bespeak that Wells‐Barnett indeed ‘theorized … but in forms quite different to from the Western [academic] form of abstract logic’ (Christian, 1987, p. 52).

Rather, her writings reflect her theorization of the realities of the US South in the mid‐to late‐19th century. She was born into an enslaved family in 1862, and she was emancipated at the age of 2. Her parents, Elizabeth and James Wells, enthusiastically engaged in political and civic activities in Mississippi, until they tragically died due to the yellow fever epidemic in 1878. Since then, she worked as a teacher, journalist who co‐owned the press Free Speech, feminist activist for Black women's rights, and Republican politician. She lived through the Reconstruction and post‐Reconstruction periods, which remained her preoccupation throughout her lifetime. In her universe, the most urgent social problem was white mobs' violence, their execution of so‐called Lynch Law, which proliferated even before the entrenchment of the Jim Crow regime.

## 2 IDA B WELLS‐BARNETT AS AN ANTICOLONIAL THEORIST

Ida B Wells‐Barnett is most cherished within the field of Black feminism. In her introduction of Wells‐Barnett's writings, Patricia Hill Collins (2002, p. 21) also presented Wells‐Barnett through the lens of Black feminism. In our humble view, the prevalent categorization of Ida B Wells‐Barnett as a Black feminist has led scholars today to focus more on the histories of Wells‐Barnett's activism (see Allen, 2022; Giddings, 2001; Schechter, 2001; Silkey, 2015) and to pay less attention to her scholarly writings of *A Red Record*, *Southern Horrors*, and *Crusade for Justice*. We suspect, this is mainly because her writings are not centred on the unique experiences of Black women or intersectional matrix of domination (see Collins, 1989). In fact, most passages in these texts focus on whites, particularly about how their violence was constitutive of the racial hierarchy and foundational structures of the US empire‐state. To clarify, we are not contesting the notion that Ida B Wells‐Barnett was a Black feminist. She was both a Black feminist and an anticolonial theorist on crime and punishment: Any *either/or* characterization (Lorde, 1984, p. 111) cannot do justice to the significance of her presence in our history. Our intention is to highlight the values of her written scholarship and to approach Ida B Wells‐Barnett as a theorist who we need to thoughtfully introduce in our social theory courses.

We have absolutely no reservations in categorizing Ida B Wells‐Barnett as an anticolonial thinker. Immersed with Wells‐Barnett's texts, both of the co‐authors of this article felt the sensation of viewing the critical moment of the Reconstruction and post‐Reconstruction periods through her worldview, and there is no better term than ‘anticolonial’ to characterise Wells‐Barnett's way of theorising the world. Foremostly, her writings poignantly unveil how the Black people—that is, the ex‐slaves, the ‘means of production’ (Jung, 2019, p. 161) that sustained US capitalism—were continuously enmeshed in the macro structures that essentially put them under colonial domination as ‘de jure and de facto practices, as less than full citizen’ (Jung, 2015, p. 68). Unsurprisingly, many of Black people (including soldiers, see Russell, 2014) opposed the US imperial warfare in Philippines and so did Wells‐Barnett as she argued, ‘Negroes should oppose expansion until the government was able to protect the Negro at home’.^2^

In her scholarly writings, she did not use the term ‘empire‐state,’ devised by Frederick Cooper (2005) a hundred‐plus years after her publications of *Southern Horrors* and *A Red Record*. She also did not use high‐level abstract languages like ‘double consciousness’ or ‘Manichaean World.’ The fact that Wells‐Barnett did not use any of those terms does not exclude her from the list of anticolonial thinkers. Dorothy Smith (1987: 69) famously argued that the usage of high‐level abstraction in academia makes the social sciences ‘extralocal’ that only helps the knowledge monopoly of a certain inner circle and that in truth objectifies local knowledges to be extracted for the powerful. Instead, Smith (ibid.: 88) suggested that we should specify our standpoint and define our specific ‘everyday world’ as problematique for scholarly enquiries. Wells‐Barnett practiced the standpoint research about one hundred years before Smith's call for reckoning the issues of subjectivity and positionality. As her everyday experiences/praxis/theory‐making (see Christian, 1987, p. 52), she negotiated with widespread horrors of lynching amongst Black people, systematically investigated the racist murders, and was involved in anti‐lynching, anti‐imperial, and feminist activism. Wells‐Barnett's standpoint was insistently bottom up, providing necessary information primarily for the antiracist activists who did not need abstract buzzwords but concrete descriptions and poignant statements.

Wells‐Barnett's scholarship and transatlantic activism (see Silkey, 2015, p. 55) was oppositional to the sociologies for the interests of empires, which Julian Go (2023, pp. 282, 290) clarified as a hallmark of anticolonial thought. The core principle of her scholarship was the ‘subaltern standpoint’ (Go, 2016, p. 173), bringing due attention to the perspectives and experiences of those repressed by the empire‐state. For example, Wells‐Barnett remarked that the crucial motivation for writing her autobiography was ‘because there is such a lack of authentic race history of Reconstruction times written by the Negro himself’ (*CJ*: 3). Due to her subaltern standpoint, Wells‐Barnett discerned how non‐governmental actors (e.g., lynch mobs and churches) and government agents (e.g., judges, sheriffs, and politicians) co‐constituted the reformation of the US empire‐state. While we elaborate on this point further in the second body section below, Wells‐Barnett's analysis of lynching as the epitome of the three foundational structures of the US empire‐state in the first body section must be understood in this context. Furthermore, Wells‐Barnett draws our attention to how crime and punishment is entangled with gender and sexuality in the processes of imperial (re)formation. While there are notable commentators on crime and punishment in empires (e.g., Agozino, 2003; Fanon, 1963; Go, 2024), gender and sexuality have been relatively undertheorised within the research programme of counter‐colonial criminology (despite Fanon, 1952). Ida B Wells‐Barnett is significant also in the sense that she was a classical anticolonial theorist who employed an intersectionality approach in her criminological analysis.

## 3 LYNCHING AND THE FOUNDATIONAL STRUCTURES OF THE US EMPIRE‐STATE

### 3.1 Lynching as the co‐re‐formation of race and gender

In Crusade for Justice (56), Wells‐Barnett described the moment that triggered her preoccupation with lynching research for years to follow: the lynching victimization of her personal friends, Thomas Moss, Calvin McDowell, and Lee Stewart on March 9, 1893, in Memphis, Tennessee. ‘This is what opened my eyes to what lynching really was … [a]n excuse to get rid of Negroes who were acquiring wealth and property and thus keep the race terrorize and “keep the nigger down”’ (ibid.). Nearly 3 months after her editorial comment on the aforementioned lynching event, the white mobs in Memphis destroyed the newspaper Free Speech that Wells‐Barnett owned. ‘They felt that the only way to restore “harmony [that is, unequal status quo] between the races” would be to get rid of the Free Speech,’ she wrote (CJ: 55).

According to the prevalent discourse at then, lynching was ‘the white southerners’ chivalrous defence of his womanhood’ (ibid.). In other words, the widespread practice of lynching was understood as southern white men's effort to save white women from Black men within the dominant white public. In this discursive framework, the categories within the entangled imperial power relations emerged as follows: (1) white men, the dominant; (2) white women, the domesticated; (3) Black men, the punishable; and (4) Black women, the sexualised.

This subsection elaborates on the aforementioned intersectional operation of power, drawing Evelyn Nakano Glenn's (2015) insight on the co‐formation of race and gender as one of the crucial foundations of the US empire. Due to the racist and patriarchal nature of US settler‐colonial conquest, ‘white women were viewed as needing to be protected by white men, particularly from the dangers posed by the primitive or perverse male sexuality of Natives, slaves, and exogenous others,’ and ‘Indian, Black, and exogenous women were viewed variously as shameless, docile, alluring, or unfeminine’ (ibid.: 69).

What Wells‐Barnett observed through her lynching research was the reformation of the foundational structure of the power relations that had emerged since the 1600s in the post‐Reconstruction period—hence this subsection's title, *co‐re‐formation of race and gender*. Central to the reformation process was the Black rapist myth, which Wells‐Barnett (*SH*: 49) called ‘the same old racket.’ In various parts of her writings, Wells‐Barnett described cases in which some white women falsely accused Black men of rape. One notable case is that of JC Underwood, the wife of a minister living in Ohio (*ARR*: 144–116). She accused William Offett, a Black man, of raping her during her husband's absence. Offett was fortunately granted a trial. There, she insisted that she did not know him, but Offett denied the rape charge, maintaining that he had entered Underwood's residence and had been intimate with her with her consent. However, ‘the sworn testimony of a minister's wife, a lady of the highest respectability,’ was sufficient evidence to sentence Offett to 15 years in prison. Interestingly, 4 years later, Underwood confessed her lie to her husband:

I sat on his lap. He made a proposal to me and I readily consented. Why I did so I do not know, but that I did is true. He visited me several times after that and each time I was indiscreet. I did not care after the first time. In fact I could not have resisted, and had no desire to resist. … I had several reasons for telling you. One was the neighbors saw the fellow here, another was, I was afraid I had contracted a loathsome disease, and still another was that I feared I might give birth to a Negro baby. (*ARR*: 116)

Discernibly, the white woman above was afraid, coerced by the intersectional power domesticating her into an immaculate housewife figure, prevalently regarded as the guardian of white racial purity. But her accomplishment of unblemished white womanhood was at the expense of punishing the Black man with whom she had romantic feelings.

Wells‐Barnett compiled another case demonstrating the triumph of the intersectional operation of power over romantic love: the lynching of Edward Coy (ARR: 117). In this case, Wells‐Barnett included two different accounts—one from the white press and another from a northern abolitionist, Albion W Tourgee. The white press simply stated that Edward Coy had assaulted a white woman, and as punishment his flesh was cut and the woman known to have been assaulted willingly set Coy on fire in front of 15,000 spectators. The abolitionist's investigation, however, revealed that the white woman had been ‘criminally intimate with Coy more than a year previous’ and that ‘Coy asked her if she would burn him after they had been sweethearting so long’ (ibid.) just before she threw the lit match on Coy's body, which had been covered in coal oil.

While the sexual activities between white women and Black men were heavily policed, white men's sexual desire for Black women was not. This impunity granted to white men is a manifestation of imperial‐intersectional power relations. Whereas the Black racist myth imposed on Black men was largely a delusion constructed by the crazy white imperialists, white men's raping had long been the reality that the enslaved/colonised women had to deal with. Due to the one‐drop rule, the offsprings of enslaved Black mothers impregnated by white men's raping were also slaves, augmenting the white masters' wealth. Due to the deprivation of indigeneity from the ‘half‐breads’ Indigenous peoples, the offsprings of Indigenous mothers impregnated by white men's raping were excluded from their rights over their land, augmenting the white invaders' wealth (Wolfe, 2006, pp. 387–388; Glenn, 2015, p. 60). This logic of US empire‐building persisted in the post‐Reconstruction period, as Wells‐Barnett (*CJ*: 59–61) observed thusly:

Such [sexual] relationships between white men and colored women were notorious, and had been as long as the two races had lived together in the South. This was so much a fact that such unions had bleached a large percentage of the Negro race, and filled it with the offspring of these unions. These children were and are known as mulattoes, quadroons, and octoroons. … I found that this rape of helpless Negro girls and women, which began in slavery days, still continued without let or hindrance, check or reproof from church, state, or press until there had been created this race within a race. … I also found that what the white man of the South practiced as all right for himself, he assumed to be unthinkable in white women. They could and did fall in love with the pretty mulatto and quadroon girls as well as black ones, but they professed an inability to imagine white women doing the same thing with Negro and mulatto men. Whenever they did so and were found out, the cry for rape was raised…

### 3.2 Lynching and the rule of difference

As much as the lynching followed by (mostly ungrounded) rape charges against Black men, the non‐lynching of rapist white men also provides evidence of the interminable nature of US imperialism, the structure of *rule of difference*. Moon‐Kie Jung's (2015: 56) advancement of the oft‐cited phrase, ‘the United States has always been an empire‐state; the United States has always been a racial state, a state of white supremacy,’ was based on his analysis of the 1857 Dred Scott‐v.‐Sandford case that epitomised the rule of difference principle.

Ida B Wells‐Barnett was astutely aware that the imperial structure of rule of difference persisted despite the Emancipation some years after the Dred Scott decision. This was especially so for the issues of crime and punishment, as she stated, ‘the punishment is not the same for both classes of criminals’ (*ARR*: 147). The impunity granted to white rapists and the death penalty of lynch law for supposed Black rapists, as discussed above, is symptomatic in this regard. ‘The miscegenation laws of the South only operate against the legitimate union of the races; they leave the white man free to seduce all the coloured girls he can, but it is death to the coloured man who yields to the force and advances of a similar attraction in white women’ (*SH*: 50–51).

Wells‐Barnett needed to deal with the dominant public having difficulty understanding the ontological presence of the criminal act of ‘rape’ committed by white men. In other words, in the dominant discourse, the wording ‘rapist’ widely invoked the image of a Black man, not a white one. Instead of the noun ‘rapist’ and the verb ‘rape,’ Wells‐Barnett deployed the wording of ‘outrage’ to refer to whites' crime of rape of Black women. In *Southern Horrors* (55), she introduced the case that three whites raped a Black woman in Baltimore, Maryland, and another two similar cases of whites' rape of Black women in Nashville, Tennessee, by using ‘outrage’ in the verb form: For example, ‘they held her escort and outraged the girl.’ Furthermore, with all of the three cases, Wells‐Barnett emphasised that those white criminals were acquitted, lightly sentenced, and bailed out, respectively, to critique the ways in which penalties were unequally imposed on white and Black peoples (for a similar usage of ‘outrage’ to critique the rule of difference, see also *ARR*: 122).

Wells‐Barnett was insistent in denouncing the lynch mob as ‘murderers’ (*ARR*: 73, 78) and ‘outlawry’ (*ARR*: 77, 151). ‘There is no doubt whatever as to the guilt of those who murdered and tortured and burned alive this victim of their blood lust,’ Wells‐Barnett (*CJ*: 74) affirmed her understanding that lynching is a criminal act of murder, witnessing a lynching case in Paris, Texas in 1893. She was well aware that the mobs' criminal acts of murder were not punished legally since ‘white men … controlled all the forces of law and order’ (*CJ*: 61), but she called for charging those white murderers within the alternative public, that is, Wells‐Barnett's readers claiming their humanitarian consciousness, for them to understand the dire situation of the rule of difference. She wrote in *A Red Record* (71–72):

not all nor nearly all of the murders done by white men, during the past thirty years in the south, have come to light, but the statistics as gathered and preserved by white men, and which have not been questioned, show that during these years more than [sic] thousand negroes have been killed in cold blood, without the formality of judicial trial and legal execution. And yet, as evidence of the absolute impunity with which the white man dares to kill a negro, the same record shows that during all these years, and for all these murders only three white men have been tried, convicted, and executed. As no white man has been lynched for the murder of colored people, these three executions are the only instances of the death penalty being visited upon white men for murdering negroes.

### 3.3 Lynching as the subversion of judge‐offender relationship

In *Crusade for Justice* (116), Ida B Wells‐Barnett recounted her speech in Liverpool, UK, in 1894, which was a response to the Black rapist myth circulated amongst white feminists in the US South:

(1) First: That all the machinery of law and politics is in the hands of those who commit the lynching; they therefore have the amending of the laws in their own hands; and that it is only wealthy white men whom the law fails to reach; that in every case of criminal procedure the Negro is punished.

(2) Second: Hundreds of Negroes including women and children are lynched for trivial offences on suspicion and in many cases when known to be guiltless of any crime, and that the law refused to punish the murderers because it is not considered a crime to kill a Negro.

Part (2) of this passage shows Wells‐Barnett's acute attention to the reality of the rule of difference, discussed extensively in the subsection above. On the other hand, Part (1) indicates that Wells‐Barnett was vigilant about the fact that lynching proceeded as if it were the execution of due punishment, although, as noted in Part (2), the executioners were the actual criminals—the lynch mobs, the ‘murderers.’ The positions of the judge and the offender are reversed in this peculiar power relation.

David Squire's (2015) commendable work clarified that lynching proceeded with Lynch Law, as if it was a due punishment over a criminal, albeit lynch mobs held no constitutional authority. He put forward that lynching can be understood, as extralegally sanctioned violence over the abandoned people—i.e., Black people—in the late‐19^th^ century US (ibid.: 144, 146). However, his reliance on Agamben's theory of homo sacer made him oblivious to power relations stemming from the foundational structures of the US empire‐state (Glenn, 2015; Jung, 2015), as much as Agamben's theory is detached from the concrete history of Western imperialisms.

In addition to the co‐re‐formation of race and gender and the rule of difference, both of which were discussed above, one of the imperial structures that were reformed after the Emancipation was what we call the *subversion of offender‐judge relationship*. On the shoulders of Du Bois, Fanon, and Rodney, Biko Agozino (2003: 4, 16, 60–61) called for our conscious recognition of slavery, genocide, and colonialism that ‘punished the innocent as though they were offenders,’ although those were the criminal acts that affected disproportionately greater numbers of peoples/cultures than the oft‐punished criminal acts like drug possession. Extending this insight further, we comprehend that such subversion of relation is a crucial nature of imperial power. The US imperialists (or settler colonialists) were the criminals who mass murdered Indigenous peoples, kidnapped Black people, and defrauded racialised peoples of their human dignity. Thinking through the canonised criminological tradition, ‘the innocent people’ (or body politic) should constitute the popular will from which magistrates draw for their exertion of punishment. However, in imperial social settings, the criminal racialisers, not the racialised innocents, constitute the popular will. Wells‐Barnett observed a reformed manifestation of this foundational structure, as she remarked thusly:

In lynching, opportunity is not given the Negro to defend himself against the unsupported accusations of white men and women. The word of the accuser is held to be true and the excited bloodthirsty mob demands that the rule of law be *reversed* and instead of proving the accused to be guilty, the victim of their hate and revenge must prove himself innocent. No evidence he can offer will satisfy the mob; he is bound hand and foot and swung into eternity. Then to excuse its infamy, the mob almost invariably reports the monstrous falsehood that its victim made a full confession before he was hanged. (Emphasis added, *ARR*: 147)

## 4 LYNCHING AND THE POST‐RECONSTRUCTION REFORM

### 4.1 Deputised death penalty as a decisive reform of the US mass‐punishment regime

Wells‐Barnett's understanding of lynching as a punishment unduly imposed on Black people by white criminals enjoying impunity, backed by the Black rapist myth, offers us a significant point of theoretical intervention. In the literature, the rise of US mass incarceration was understood as a state‐engineered fix to tackle the structural crisis of capitalism in the 1970s (Gilmore, 2007) or as a state's backlash against the Civil Rights movements through welfare programs featuring social workers' widespread practices of profiling poor Black people with criminality in the 1960s (Hinton, 2017). Although they did not trace the origin of the US mass‐punishment regime in the late‐19th century, we agree with one theoretical insight that the two aforementioned authors provide: The US mass‐punishment regime deviates from the Weberian model of direct state violence, as the crucial criminalising actors are loosely linked to the central state but not directly commanded by the statist directives. Khalil Muhammad's (2010) work exceptionally traced the criminalisation of Black people in the late‐19th century, and his work also features the actors outside the central state's chain of command; that is, social scientists equipped with the incipient form of statistical methods that so‐to‐say ‘proves’ the Black inferiority with disproportionately high crime rates of Black population. Muhammad's work is indeed a good complement to our interpretation of Ida B Wells‐Barnett. In northern industrialised cities like Chicago, New York, and Philadelphia, social scientists played their part in reaffirming the empire's racial hierarchy. In the US South, although lynching also occurred in some northern towns intermittently, the lynch mobs' violence played one of the most significant parts that governed the daily lives of both whites and Black people. One remarkable work that demonstrates a similar understanding on the rise of US mass punishment regime is by historian Kelly Lytle Hernández (2017: 1, 197): The widespread practice lynching was one of many forms of violence that white imperialists deployed as one of the ‘eliminatory options’ against the troubling segments amongst the racialised since the settler colonial expansion, and hence it constitutes the historical ‘upriver’ of the mass incarceration later developed in the late‐1960s.

While we share the understanding that eliminatory violence is a structure, not an event, with Hernández (2017; see also Wolfe, 2006), the part that we wish to highlight is how the structure became decisively reproduced despite the nominal transformation, that is, the abolition of slavery, via the constitutive agency of the white lynch mobs. Wells‐Barnett enlightens us that the rise of lynching is a crucial page of US history, showing the late‐19th century reformation of the US mass‐punishment regime: The foundational structure of the US empire‐state manifested in the form of genocide and slavery (see Agozino, 2003; Glenn, 2015), and it became *reformed*—not *transformed*—in the late‐19th century into the prevalence of lynching, as the deputised mass death penalty.

In terms of the deputised nature, Wells‐Barnett raised the following two occasions (*ARR*: 147–148):

[1] Mayor Trout, of Roanoke, Virginia, called out the militia in 1893, to protect a negro prisoner, and in so doing nine men were killed and a number wounded. Then the mayor and militia withdrew, left the negro to his fate and he was promptly lynched. The businessmen realized the blow to the town’s financial interests, [and] called the mayor home. The grand jury indicted and prosecuted the ringleader of the mob. They were given light sentences, the highest being one of twelve months in State prison. They day he arrived at the penitentiary, he was pardoned by the governor of the State.^3^

[2] In South Carolina, in April, 1893, Gov. Tillman aided the mob by yielding up to be killed, a prisoner of the law, who had voluntarily placed himself under the Governor’s protection. Public sentiment by its representatives has encouraged Lynch Law, and upon the revolution of this sentiment we must depend for its abolition.

In the first case, the incredibly light penalty given to the lynch mob leader reminds us of the imperial structure of rule of difference. An even more striking fact is that the mayor ‘withdrew’ the protection of the prisoner so that lynch mobs could ‘promptly’ murder the Black prisoner. This deliberate allowance of lynching is unmistakably clear in the second case. The governor ‘aided the mob by yielding up’ the Black prisoner to be murdered. Crucial here is that, as Wells‐Barnett observed, the racist violence was deputised to lynch mobs' execution. The punishment was not just to the extent of imprisoning the Black ‘criminals,’ so‐to‐say, but that of letting lynch mobs murder them so that the murdered Black people remain unaccounted for by the formal judicial system. Upon the operation of deputised violence, the lynch mobs were affirmed of their impunity, as shown in the first occasion.

Wells‐Barnett's observation above reveals the US racial‐penal regime's structural nature over a long durée, which the previous scholarship (Gilmore, 2007; Muhammad, 2010; Hinton, 2017; but Hernández, 2017) overlooked. We must remind ourselves that mass incarceration, much debated since the 1960s, is essentially a social phenomenon of mass punishment, and hence, our observation should be more geared towards the primitive forms of punishment, that is, lynching, rather than literal incarceration, carceral capacity, and conviction. The visceral punishment in the form of lynching was not intended to put the ex‐enslaved people under the formal judicial system but to murder them en masse as ostracised bodies from the system that exclusively serves imperial citizens, not the racialised ex‐enslaved.

### 4.2 The lynchpin of the structural reformation of the US empire‐state

Precisely for the point above, we argue that the proliferation of lynching embarked on the structural reformation of the US empire‐state, and Ida B Wells‐Barnett analysed this moment. The following is her historical account of the post‐1865 period, based on her systematic investigation of the discursive changes in the white press, which appeared in *A Red Record* (71–73).

The slave was rarely killed, he was too valuable … In slave times the Negro was kept subservient and submissive by frequency and severity of scourging, but, with freedom, a new system of intimidation came into vogue; the Negro was not only whipped and scourged; he was killed. … [T]here distinct excuses have been made. The first excuse given to the civilized world for the murder of unoffending Negroes was the necessity of the white man to repress and stamp out alleged ‘race riots.’ … It was always a remarkable feature in these insurrections and riots that only Negroes were killed during the rioting and that all the white men escaped unharmed. … Then came the second excuse, which had its birth during the turbulent times of reconstruction. By an amendment to the Constitution the Negro was given the right to franchise, and, theoretically at least, his ballot became his invaluable emblem of citizenship. … The government which had made a citizen found itself unable to protect him. … Scourged from his home; hunted through the swamps; hung by midnight raiders, and openly murdered in the light of day, the Negro clung to his right of franchise with a heroism which would have wrung admiration from the hearts of savages. … [T]he murderers invented the third excuse—that Negroes had to be killed to avenge their assaults upon women. … With such unanimity, earnestness and apparent candor was this charge made and reiterated that the world has accepted the story that the Negro is a monster which the Southern white man has painted him.

Wells‐Barnett discovered that, prior to the well‐known excuse—‘the same old racket’ (*SH*: 49) of white men saving white women from Black men—two additional naked excuses existed. As Wells‐Barnett observed, those two excuses were to subdue the Black sovereignty, that is, their rights to defend themselves and vote. Those rights were emblematic of citizenry dignity, which the enslaved Black folks never had a glimpse of prior to 1865. In this context, Wells‐Barnett's temporal focus on the Reconstruction to post‐Reconstruction period is noteworthy. As she noted, the enslaved Black people were ‘too valuable’ to kill since they were ‘means of production’ (Jung, 2019, p. 161) of early US capitalism. After those ‘means of production’ (ibid.) gained nominal citizenship, lynching proliferated to subdue their sovereignty.

Again, through the Reconstruction and post‐Reconstruction period, the US empire‐state experienced a structural *reform*, not a total transformation but some configurational adjustments of its racial structure. The crux of this reform process was the agency of white presses, opinion elites (including religious leaders and politicians), and lynch mobs, which ultimately constituted the structure(s) of the US empire‐state. Here, helpful is Moon‐Kie Jung's (2015) dissection of the US empire‐state. For him, the empire is composed of layers of structures sustained by myriads of actors' agency—structure and agency are not antithetical but mutually constitutive. Rectifying Sewell's (2005) conceptualisation, Jung (2015: 27–32) advanced that not only actions and materials but also knowledge, emotion, and culture are resoundingly actual (not virtual) components of structure, which is pertinent to the racism structure of the US empire‐state.

The racism structure could have been disarticulated due to the 1865 Amendment, but it became rearticulated into the reformed racism structure through the proliferated lynching, as researched by Ida B Wells‐Barnett. Also, as discussed above, Wells‐Barnett understood the deputised violence of lynching to coincide with the discursive criminalisation of the freed Black people. Her second usage of the word ‘outrage’ in *A Red Record* (116–117, emphasis added) pertains to this understanding: She analysed a newspaper article on Lillie Bailey, a seventeen‐year‐old white mother of a Black baby, who did ‘not divulge the name of the man who has left such black evidence of her disgrace’ and hence did not ‘reveal fearful depravity’ of Black rapists, ‘the evidence of a rank *outrage*.’ She comprehended this anecdote as a case of manufacturing white's outrage—a noun for indignant emotion—against Black people by invoking the Black rapist myth, again, ‘the same old racket’ (*SH*: 49). Furthermore, this outrage, grounded or ungrounded on fact, as it circulated via cross‐citation amongst opinion elites (including religious leaders, white intellectuals, judges) within white presses, incessantly gained power. Wells‐Barnett condemned this circulation of outrage thusly:

Dr. Hoss, editor of the leading organ of the Methodist Church South, published in its columns that it was his *belief* that more than three hundred women had been assaulted by negro men within three months. When asked to prove his charges, or give a single case upon which his ‘belief’ was founded, he said that he could do so, but the details were unfit for publication. No other evidence but his ‘belief’ could be adduced to substantiate this grave charge, yet Bishop Haygood, in the Forum of October, 1893, quotes this ‘belief’ in apology for lynching, and voluntarily adds: ‘It is my *opinion* that this is an underestimate.’ The ‘opinion’ of this man, based upon a ‘belief,’ had greater weight coming from a man who has posed as a friend to ‘Our Brother in Black,’ and was accepted as authority. An interview of Miss Frances E. Willard, the great apostle of temperance, the daughter of abolitionists and a personal friend and helper of many individual colored people, has been quoted in support of the utterance of this calumny against a weak and defenseless race. In the New York Voice of October 23, 1890, after a tour in the South, where she was told all these things by the ‘best white people,’ she said: ‘The grogshop is the Negro’s center of power. Better whisky and more of it is the rallying cry of great, dark‐faced mobs. The colored race multiplies like the locusts of Egypt. The grogshop is its center of power. The safety of woman, of childhood, the home, is menaced in a thousand localities at this moment, so that men dare not go beyond the sight of their own roof‐tree.’ These charges so often reiterated, have had the effect of fastening the odium upon the [Black] race of a peculiar propensity for this foul crime. (*ARR*: 133, emphasis added)

Amy Wood (2009) helpfully theorised the witnessing of lynching in the US South, in person or via press coverage, was an exertion of the white spectators' agency, their social‐psychological agency to keep the pre‐1865 racial hierarchy. The excerpt above exhibits that Wells‐Barnett also observed the discursive formation of hate against Black people through the spectatorship mediated via white presses. This discursive formation of the whites' popular will (or collective consciousness) to mass murder or at least to feel hostility against Black people was the reconstituted configuration of the imperial structure of racism.

In this context, as noted in *A Red Record* (71–73) introduced earlier, with the ‘evolution’ of lynching excuses, racial hate became gradually entrenched in not only the white public sphere (e.g., lynching sites or presses) but also the whites' private sphere. The hate was initially about Black people's capability of defence and voting, but from around the 1890s, the hate evolved to target Black people's sexuality. We can reasonably guess that this life‐world colonization was a consequence of the hate circulation within white presses. In other words, the white womanhood domesticated to keep their race pure and to participate in murdering Black ‘rapists’ with their falsehood, discussed in the earlier section, was an effect of this hate circulation. By the 1890s, sustained by this racial hate, the presence of lynching tied the private and public realms in the US South, regardless of racial division.

The lynching of Black people was the *lynchpin* that the US imperialists relied on to keep their white selfhood intact, as it was an extraordinarily necessary part of US Southern lives as the assurance of the white supremacist social order in the post‐Reconstruction period. Patterson (1999) poignantly noted that the Southerners' impulse to suppress the burgeoning self‐sovereignty of emancipated Black people necessitated meticulous rituals to reaffirm non‐humanity of the lynched Black people in the proceedings. Under this reformed structure, whites' subjecthood was conditioned on the mass deaths of the emancipated Black people.

At the same time, the widespread lynching was the *lynchpin* of the US imperial structure in the other way, too. The barbarous mass murder had to be concealed or justified, and otherwise the status of the US *civilization*—internationally known as being built by whites—could be jeopardised by the international public, especially amongst fellow whites in Europe (see Silkey, 2015, pp. 6–7). In this context, precisely as much as the white elites were concerned about their civilisational status, Wells‐Barnett's anti‐lynching campaigns in Britain (1893–1894) and in the US South troubled the lynchpin. In fact, Wells‐Barnett published *A Red Record* in 1895, printed in the form of a pamphlet, precisely with the intention of putting the civilisational status of the US whites under indictment. In the last chapter of the pamphlet, Wells‐Barnett stated thusly:

[R]emembering that after all, it is the white man’s civilization and the white man’s government which are on trial. This crusade [i.e., Wells‐Barnett’s anti‐lynching activism] will determine whether that civilization can maintain itself by itself, or whether anarchy shall prevail; whether this Nation shall write itself down a success at self government, or in deepest humiliation admit its failure complete; whether the precepts and theories of Christianity are professed and practiced by American white people as Golden Rules of thought and action, or adopted as a system of morals to be preached to heathen until they attain to the intelligence which needs the system of Lynch Law. (*ARR*: 149, The authors' interpolation in the bracket.)

Wells‐Barnett was well aware that a new rhetorical tactic to criminalise lynch mobs was necessary, as she noted in her 1893 publication of Southern Horrors (65, emphasis in original), ‘*[b]ut they did not punish the lynchers*.’ Thus, for Wells‐Barnett, this possibility of white‐charging‐white practices was instrumental in her anti‐lynching politics. The ‘radical statistics’ (Brubaker, 2022) provided in *A Red Record* (142–146, especially) was precisely to spread the alternative knowledge not only for Black people but also for European whites about the barbarity of the US.

If the Southern people in defense of their lawlessness, would tell the truth and admit that colored men and women are lynched for almost any offense, from murder to a misdemeanor, there would not now be the necessity for this defense. But when they intentionally, maliciously and constantly belie the record and bolster up these falsehoods by the words of legislators, preachers, governors and bishops, then the Negro must give to the world his side of the awful story. (*ARR*: 74)

## 5 WELLS‐BARNETT'S PLACE IN ANTICOLONIAL SOCIOLOGY

In sum, Wells‐Barnett discerned the early formation of the racist mass punishment regime by observing the proliferation of lynching. We argue, in doing so, she apprehended the moment of post‐Reconstruction structural reformation of the US empire‐state. The foundational social structures of the US empire‐state include the co‐re‐formation of race and gender, rule of difference, and subversion of judge‐offender relationship (see Agozino, 2003; Glenn, 2015; Jung, 2015). From this vantage point, we aim to highlight how Wells‐Barnett's theory offers broader implications to anticolonial/postcolonial sociology, particularly through her subaltern standpoint, attention to the role of non‐state actors, and her commitment to intersectional analysis.

First and foremost, she adopted the ‘subaltern standpoint’ (Go, 2016, p. 173) of the formerly enslaved in theorising the local realities of the US South in the post‐Reconstruction period, where freed Black people were subjected to the pervasive terror of lynching. Although she was rooted in her historical situation in Memphis, TN (and later in Chicago, IL), her theorisation (Christian, 1987, p. 52) yielded crucial insights relevant to the transnational scope of the US empire‐state and other imperial formations. For example, it is telling that Fanon shared a similar understanding of the subverted offender‐judge relationship: While it was the French colonisers who committed the crimes of ‘looting, raping, and starving to death’ (Fanon, 1963, p. 15), they assumed the position of criminal justice authority. Fanon also observed that it is not the rule of law but the rule of difference that prevailed in colonial situations, noting that ‘not a single Frenchman has been brought before a French court of justice for the murder of an Algerian’ (ibid.: 50). Despite the similarity in their understanding of the two imperialisms, Wells‐Barnett did not necessarily devise ‘generalisable’ concepts but aimed to archive the historical realities of her time/space.

In this regard, Wells‐Barnett offers anticolonial/postcolonial sociologists an alternative approach to conceiving ‘broader implications’ of theories. Many sociologists draw concepts from social theorists without reflecting on their historical contexts, assuming the concept in question possesses a *generality* or applicability that transcends time and space. Rather than seeking for generality, we advocate for considering the *conjunctural relevance* of concepts or propositions. Wells‐Barnett apprehended the reformation of the US empire‐state through analysing the three foundational structures tethered to the proliferation of lynching, specifically in the post‐Reconstruction period. This should itself be regarded as her theorised observation of a historical conjuncture (Steinmetz, 2014, p. 92), which can be contextualized in relation to other conjunctures of US imperialism across different times and spaces. As discussed earlier, while prototypical crime statistics contributed to the criminalisation of Black people in northern industrial cities (Muhammad, 2010), in the US South, lynching practices played a decisive role in reforming the racial hierarchy. The rise of lynching as a form of mass punishment, in turn, prefigured the racist mass incarceration from the 1960s (see Hinton, 2017). Discussing the implications of theories in terms of their conjunctural relevance is as fruitful as, if not more so than, expecting generality from them.

Second, because Wells‐Barnett assumed the standpoint of ordinary Black folks who were freed from enslavement but remained excluded from the judicial system, she was compelled to closely examine the role of non‐state actors (e.g., lynch mobs) in co‐constituting the reformation of the US empire‐state. Observing the US South, where Black folks—including Wells‐Barnett and her family—had been enslaved the most, she recognised lynch mobs as actors holding historical *agency* rearticulating the US racial hierarchy, which had been momentarily disrupted by the Emancipation. The agentic role of non‐state actors in constituting imperialism has been increasingly discussed in the anticolonial/postcolonial sociology literature (e.g., Jung, 2015; Mbembe, 2019, p. 70; Kim, 2023). Had Wells‐Barnett's theory received due attention, sociologists in general—not just anticolonial sociologists—might have paid greater attention to the history‐making agency of non‐state actors. This significance of non‐state actors suggests that our everyday life, even without direct state intervention, is a site of power struggles. Hence, everyday racism is as potent as the US empire‐state's overseas militarism, and thence our contestation against a racist next door carries historical significance. Also, as lynch mobs were making history, Ida B Wells‐Barnett was also making history through her agency, that is, scholarship and advocacy, challenging the wicked reformation of the empire. Perhaps, we should seek decolonial potential in our everyday actions rather than in spectacular events.

Lastly, we should draw a lesson from Wells‐Barnett's intersectional analysis of lynching and her unwavering commitment to speaking the truth as a Black woman thinker. Sociologists have taken a long detour before arriving at a point where they are finally prepared to listen to Black women (see Luna et al., 2024). It was only from the 1980s onward that sociologists, influenced by Black women scholars (especially from outside the discipline), began to vaguely understand the multidimensionality of oppressions and privileges and the differential experiences by vary positions within the matrix of domination (see Collins, 1989). Strictly considering the historical timeline, Wells‐Barnett should be recognised as a classical social theorist, alongside WEB Du Bois, anchoring sociology with shared problematiques and theoretical lexicons. She has already been speaking to us. Wells‐Barnett could already have been recognised as a foundation thinker for intersectional analysis, had we listened to her. Better later than never; just as Wells‐Barnett approached lynching with an intersectional sensibility, we can learn to analyse any socio‐historical phenomenon through an intersectional lens. In this context, Jordanna Matlon's (2024) advocacy for including gender in analyses of racial capitalism is a commendable development. In fact, Evelyn Nakno Glenn (2015), Vrushali Patil (2022), and Jyoti Puri (2021) have long dedicated their careers to examining the intersections of empire, race, gender, and sexuality. We should contribute to this programme further.

Confronting the oppressions structured within the US empire‐state, Ida B Wells‐Barnett offers crucial—though often overlooked—theoretical insights as an anticolonial theorist, as discussed above, particularly on crime and punishment (see also Agozino & Ducey, 2020). We do not mean to claim, however, that our interpretation is the only correct way to understand her scholarship, or that Wells‐Barnett is solely an anticolonial theorist. We reiterate that any form of *either/or* characterisation (Lorde, 1984, p. 111) does an injustice to our history with her. This is an invitation for further research on Ida B Wells‐Barnett. Open the pages of *Southern Horrors*, *A Red Record*, and *Crusade for Justice*. You will liberate yourself and those around you.

## CONFLICT OF INTEREST STATEMENT

The author declare no conflicts of interest.

## Data Availability

Data sharing not applicable to this article as no datasets were generated or analysed during the current study.

## ENDNOTES

^1^
We use the acronym thusly: ARR as *A Red Record*; SH as *Southern Horrors*; and CJ as *Crusade for Justice*, referring to the volumes edited by Royster (Wells‐Barnett, 2016) and Duster (Wells‐Barnett, 2020).

^2^
Cleveland Gazette, 7 January 1899.

^3^
This part followed the correction provided by Jacqueline Royster.
